# The Use of Lavender (*Lavandula angustifolia*) Essential Oil as an Additive to Drinking Water for Broiler Chickens and Its In Vitro Reaction with Enrofloxacin

**DOI:** 10.3390/ani11061535

**Published:** 2021-05-25

**Authors:** M. Adaszyńska-Skwirzyńska, D. Szczerbińska, S. Zych

**Affiliations:** 1 Laboratory of Poultry Science, Department of Monogastric Animal Sciences, West Pomeranian University of Technology Szczecin, Janickiego Str. 29, 71-270 Szczecin, Poland; danuta.szczerbinska@zut.edu.pl; 2 Labo-Wet Laboratory, Pyrzycka 9A Str., 70-892 Szczecin, Poland; maxbiotech@interia.pl

**Keywords:** essential oils, lavender (*Lavandula angustifolia*), broiler chicken, enrofloxacin

## Abstract

**Simple Summary:**

Essential oils can stimulate the growth and functioning of the body, which translates into both chickens’ health and enhanced production parameters. Given the increasing restrictions imposed on poultry production in terms of food safety, it seems appropriate to look for the use of new, natural substances to be applied in poultry production. The article presents the results of the study of lavender essential oil in this context, with a particular focus on its antimicrobial and immunostimulatory properties. The purpose of the study was to determine the influence of lavender essential oil (added to drinking water at concentrations of 0.4 mL/L) on the production parameters and selected blood parameters in broiler chickens and to assess the lavender oil in vitro reaction combined with a commercial antibiotic, enrofloxacin, against *Escherichia coli.* The results of the experiment showed that the lavender essential oil exerts antimicrobial and antioxidant activity and it also showed a positive effect on the production results of broiler chickens.

**Abstract:**

Biological activity of lavender essential oil is a property that can potentially find an application in poultry nutrition. Nowadays, the use of bioactive compounds is encouraged in many areas of industry and agriculture, since these substances have similar properties as withdrawn antibiotic growth promoters. Additionally, antibiotic resistance bacteria are one of the most important current threats to animal health. The purpose of the study was to determine the influence of lavender essential oil on the production parameters and blood parameters in broiler chickens and to assess the lavender oil’s in vitro reaction in a combination with enrofloxacin towards *Escherichia coli.* One-day-old non-sexed chicks (Ross 308) were divided into three experimental groups, each consisting of 100 individuals (five replicate of 20 boiler chicken each). The chickens in the control group received drinking water with no addition of lavender essential oil. In the experimental groups, lavender oil was added to the drinking water at a concentration of 0.4 mL/L, in the LEO_1–42_ from 1 to 42 days of age and the LEO_22–42_ group from the 22 to 42 days of age. The chickens’ body weight, feed consumption, water consumption, deaths and elimination due to health reasons were determined in the experiment. On day 42 of the chickens’ lives, blood samples were collected based on which selected parameters were identified. An in vitro experiment of lavender oil in combination with enrofloxacin was investigated with a checkerboard method. The results of the experiment showed the antimicrobial and antioxidant activity of lavender essential oil and its positive effect on the production results of broiler chickens. The study results proved that the addition of lavender oil positively impacted the chickens’ final body weight and feed conversion ratio (*p* < 0.01). No differences were observed between the groups for water consumption, death rate and the examined biochemical and immunological blood serum indices. Lavender essential oil was demonstrated to increase the blood serum’s total antioxidant status. A synergistic reaction in vitro was observed for lavender oil combined with enrofloxacin against resistant strains of *Escherichia coli*. Based on our study, a health-promoting effect of adding LEO to water for broiler chickens was found. Moreover, in vitro studies indicate a significant effect of lavender essential oil on the inhibition of the resistant strains of *Escherichia coli* growth and synergistic reaction with enrofloxacin.

## 1. Introduction

Contemporary broiler chickens are characterised by a gradually increased growth rate but are also more susceptible to different disorders and diseases, including but not limited to alimentary tract diseases, such as colibacteriosis [1]. Herbs deserve special attention because they have become more prevalent in feed mixes and supplementation preparations due to the content of valuable biologically active substances, e.g., essential oils (EOs) [2,3,4,5,6,7]. EOs are characterised by a multi-directional action [3,7,8]. Their use in diet improves the feeds taste and aroma, increases the feed intake, regulates digestive function, modifies the microbiota or the animal’s gastro-intestinal tract, consequently improving growth and feed use, which is particularly important for the increasing cost of feed [3,4,6]. Lavender essential oil (*Lavandula angustifolia*—LEO) is among the EOs that deserve greater focus. It has antibacterial, antifungal, antioxidant and immunostimulating action [3,4,5]. The main LEO’s ingredients include linalool, linalool acetate, lavandulol and γ-terpinol, which reveal a broad scope of biological and pharmacological action [6,7,8]. On the one hand, LEO can be characterised by an activity that limits the development of pathogenic bacterial flora, and on the other hand, it can stimulate the growth of the beneficial microbiota [4,5,6]. Intensive farming means exposing broiler chickens to stress resulting from feed changing, transport and stocking density [9,10]. The factors mentioned above may negatively impact the humoral and cellular immunological response, which means that such pathogenic bacteria can colonise the broiler chickens’ alimentary tract as Salmonella, *Escherichia coli*, *Campylobacter jejuni* and *Clostridium perfringens* genera [1,9]. Colibacteriosis, being one of the most often diagnosed diseases in slaughter chickens, is specified as being among the biggest problems in contemporary animal production [1]. Enrofloxacin, an antibiotic from the fluoroquinolone group, is commonly used for treating infections caused by *E. coli.* Antibiotics exert selective pressure towards increasing the count of antibiotic-resistant bacteria. Studies aimed at improving antibiotic therapies efficiency shall be intensified in light of the spreading problem of antibiotic resistance and increasing genetic variability of pathogenic bacteria that have played an increasingly important role in intensive poultry production, including but not limited to multi-factor aetiology diseases. It is also necessary to reduce synthetic antibiotics’ use to the necessary minimum and replace them with natural phytobiotics that can offer specific prevention against some bacterial infections. Concerning the above, studies were carried out to determine the influence of the natural LEO addition to the chickens’ drinking water on the growth performance parameters (body weight—BW; average daily gain—ADG; feed intake—FI; feed conversion ratio—FCR; European Broiler Index—EBI), the level of biochemical (alkaline phosphatase—ALP; alanine aminotransferase—ALT; aspartate aminotransferase—AST; cholesterol; glucose; total protein; triglyceride; uric acid), immunological (IgA, IgG, IgM) and antioxidant (Total Antioxidant Status—TAS) indices of the blood serum. Additionally, this study aimed to verify the existence of in vitro reaction effect of LEO combination with antibacterial drug enrofloxacin.

## 2. Materials and Methods

### 2.1. Essential Oil

A commercial LEO (Natural Lavender Oil, Avicenna-Oil, Wrocław, Poland) was used for the study. The essential oil manufacturer (Avicenna-Oil, Wrocław, Poland) reported that the LEO was isolated by means of typical distillation with water vapour. Chromatographic analysis of the essential oil was performed using an Agilent 6890 N gas chromatograph with a 5973 N mass selective detector and a 7683 Series Injector [4,6]. The LEO ingredients were described in our previous study—the main ingredients included linalool 35.17% and linalool acetate 46.25% [4,6].

### 2.2. Broiler Chickens’ Experiment

#### 2.2.1. Growth Performance Parameters

The experiment was carried out on a commercial farm (Żabówko, Poland) on 300 non-sexed Ross 308 broiler chickens. The broiler chickens were obtained from broiler breeding flocks, 40 weeks of age, purchased from a commercial hatchery (Park Drobiarski Sp. z o.o., Śmiłowo, Poland). The transport distance from the hatchery was 150 km. One-day-old chicks were randomly divided into three experimental groups, each consisting of 100 broiler chickens (five replicates of 20 broiler chicken each). The control group of broiler chickens received drinking water with no LEO addition throughout the 42-day rearing period. In the LEO_1–42_ and LEO_22–42_ groups, LEO was added to drinking water at 0.4 mL/L (for a 6 h/day—the mean time of WI with LEO throughout the production cycle): In the LEO_1–42_ group, from day 1 to 42 day, and in the LEO_22–42_ group, from day 22 to 42. The broiler chickens were provided with water in reversible waterers on a 5 L manual reservoir tank (in each pen). Once all the LEO-infused water was consumed, the reservoir tanks were replaced with new ones containing clean water without the addition of LEO. The addition of LEO to water from d 22 is justified by the influence of the operation for intensive rearing conditions, which intensifies in the second rearing period (inter alia deterioration of the quality of litter, as well as an increase—in the quantity of conditionally pathogenic bacteria, the concentrations of ammonia in the air and the transmitted heat from the body to the environment). The experimental broiler chickens were kept in the same room with a commercial flock for 42 day, on wheat straw, at a stocking density of 14 broiler chickens/m^2^. The environmental conditions for all groups were standardised and compliant with Ross 308 [11]. At the initial stage of rearing (day 1–2), the mean temperature in the shed was 32–33 °C and decreased in subsequent periods as follows: 30 °C (day 3), 28–30 °C (day 4–7), 26–28 °C (day 8–14), 24–26 °C (day 15–21), about 22 °C (day 22–42). The average humidity ranged from 50% at the beginning to 70% in the final stage of the cycle, increasing gradually from week to week. In addition, the broiler chickens were vaccinated against Newcastle disease virus (NDV) and infectious bronchitis virus (IBV) after hatching and on d 11 respectively; and vaccinated with the infectious bursal disease (IBD) vaccine on d 17 according to the routine immunisation programme. The broiler chickens were fed ad libitum with diets from a commercial feed production plant (Polskie Zakłady Zbożowe, Wałcz, Poland): Starter (from 0 to 12 day of rearing), grower I (from 13 to 22 day of rearing), grower II (from 23 to 32 day of rearing) and finisher (from the 33 to the 42 day of rearing). The ingredients and nutritive value of the diets are summarised in Table 1. The broiler chickens’ BW (on day 1, 7, 14, 21, 28, 35 and 42 days of age), daily FI and WI and the number of dead birds were recorded in the experiment. All measurements were taken between 08.00–09.00 in the morning. In the experiment, the growth performance parameters were evaluated: ADG, FCR, EBI and chickens’ survival rate was determined based on the results.

#### 2.2.2. Animal Welfare Statement

The authors confirm that they have followed EU standards for the protection of animals used for scientific purposes. The experiment was carried out upon the approval of the Local Ethical Committee for Experiments on Animals in Szczecin, Poland (resolution No. 19/2015 of 22 May 2015).

#### 2.2.3. Blood Parameters

In order to determine biochemical, immunological and antioxidant indices, blood samples were collected from the left-wing vein from ten random broiler chickens in each group (two bird/replicates) on day 42 of the age of rearing. These broiler chickens were weighed and fasted 8 h of feed deprivation before sampling. The blood samples were carried out between 08.00–09.00 in the morning. The blood samples were centrifuged for five minutes at 15,000 rpm to obtain the blood serum. The serum was stored and monitored under refrigerated conditions (+5 ± 0.5 °C) until the analysis started. The samples temperature at the identification time amounted to +18 ± 1 °C. Blood biochemical indices concentration was performed using a biochemical analyser (VetTest 8008 from Idexx Laboratories, Inc., Westbrook, ME, USA). The following biochemical indices were analysed: Alkaline phosphatase (ALP), alanine aminotransferase (ALT), aspartate aminotransferase (AST), cholesterol, glucose, total protein, triglyceride and uric acid. The serum samples were also used to measure concentrations immunoglobulins (IgA, IgG and IgM isotypes), using hen specific immunoglobulins ELISA quantitation kits (Bethyl Laboratories, Montgomery, TX, USA). The values were determined with a synergy fluorescence, luminescence and absorbance reader from BioTek Instruments (Winooski, VT, USA), with a measured absorbance at 450 nm [12]. The randox colourimetric test was employed to determine the TAS, involving ABTS (2,2′-azino-bis (3-ethylbenzothiazoline-6-sulfonic acid)) reaction with peroxidase. The determination was performed using a Pentra 400 biochemical analyser from Horiba ABX (Montpellier, France). The absorbance was used and measured at 660 nm. The method was calibrated with the vitamin E analogue, known as Trolox equivalent, and the results are expressed in mmol/L. All serum samples were tested in triplicate.

### 2.3. In Vitro Experiment

#### 2.3.1. Antioxidant Activity

The antioxidant activity of LEO was evaluated by the measurement of the capacity of trapping radical 1,1-diphenyl-2-picryhydrazyl—DPPH (Alfa Aesar^TM^, Lancashire, UK). The stock solution of LEO was prepared in methanol to achieve a concentration of 1 mg/mL. Dilutions 1000, 500, 250, 125, 62.5, 31.25,15.62 and 7.81 μg/mL were prepared by the serial dilution method. The diluted solutions (1 mL each) were mixed with 1 mL of methanolic solution of DPPH (1 mg/mL). After 30 min incubation in darkness at room temperature (22 °C), the absorbance was recorded at 517 nm using a UV-VIS spectrophotometer (GBC Scientific Equipment, model 916). Synthetic antioxidant reagent 3-tert-butyl-4-hydroxyanisole—BHA (Sigma-Aldrich, number catalogue: PHR1306, St. Louis, MI, USA) was used as the positive control. The inhibition of DPPH by BHA was also analysed with the same concentration for comparison [13]. Inhibition free radical DPPH in percent (I%) was calculated in the following way:

(1) $$Inhibition\%=\frac{A_{blank}-A_{sample}}{A_{blank}}\times 100$$

where *A_blank_* is the absorbance of the control reaction (containing all reagents except the test compound), and *A_sample_* is the absorbance of the test compound. The variance of antioxidant activity for the LEO and BHA (percentage of inhibition and index IC_50_) were calculated. IC_50_ values were estimated from the percentage of inhibition versus the concentration plot using a non-linear regression algorithm.

#### 2.3.2. Antibacterial Activity—Microdilution Checkerboard Method

The isolates originated from the strain collection (Labo-Wet Laboratory, Szczecin, Poland) isolated between 2014 and 2016 from chicks from several subsequent placements (Żabówko, Poland). The strains were banked in ViaBank™ strain archiving systems (BioMaxima, Gdańsk, Poland). A model strain of *E. coli* (KwikStik™, Microbiologics, St. Cloud, MS, USA) and five *E. coli* isolates from omphalitis, and yolk sac cases in one-day-old chicks were selected for the analysis of sensitivity to enrofloxacin and LEO; the isolates were characterised by variable sensitivity to a standard absorbent paper disc of enrofloxacin 5 μg in the disc diffusion method. The antibacterial action of LEO and enrofloxacin (100 mg/mL; Bayer Animal Health, Leverkusen, Deutschland) were determined by the means of the Minimum Inhibitory Concentration (MIC) microdilution assay and verified according to CLSI recommendations (Clinical and Laboratory Standard Institute, 2002) Protocol M7-A6. The dilutions were performed using DMSO (POCH, Gliwice, Poland). The bacteria were inoculated by the reductive culture into a Columbia Blood agar with 5% of sheep blood (Oxoid Limited, Hampshire, United Kingdom) medium and incubated for 24 h at 37 °C. After 24 h, 2 to 5 typical colonies of the working strain culture were collected and suspended in an isotonic, sterile 0.85% NaCl solution.

Next, the optical density was measured with a density meter (measurement deviation of ±0.1, in the 0.00–3.00 range according to McFarland’s scale), causing turbidity of 0.5 MF, which corresponded to the average quantity of 1.5 × 10^8^ CFU/mL. All identifications were made on individually packed, sterile, divided, 96-well polystyrene titration plates with flat bottoms. A series of two-fold dilutions of each antimicrobial agent were prepared in Mueller–Hinton broth Oxid Limited, Hampshire, United Kingdom ) ranging from 0.001–100 μg/mL for enrofloxacin, while for LEO, the concentration gradient was 0.005–50% *v*/*v*. At the next stage, 0.005 mL of bacterial was added to each well (at final concentrations of 10^5^CFU/mL in the well). In order to exclude an inhibitory effect of DMSO and possible contaminations, positive and negative control assays were also performed. The resulting checkerboard contains a combination of a concentration gradient of two antimicrobial agents, wherein wells that contain the highest concentration of each agent were located at opposite corners of a plate. The plates were sealed and incubated at 37 ± 1 °C for 24 h. At the end of the incubation, MIC was defined as the lowest concentration that did not result in any visible growth of the bacterial strains compared to their growth in the control wells. MIC determinations were realised in triplicate in three independent assays.

Additionally, the results were recorded as the absorbance of individual plate wells using a 650 nm wavelength filter in a BioTek Elx800 microplate reader. MIC data of the LEO and enrofloxacin were converted into Fractional Inhibitory Concentration (FIC) and defined as the antimicrobial concentration in an inhibitory concentration with a second compound to the concentration of the antimicrobial by itself [14]. In the combination assays, the checkerboard procedure described by Rosato et al. [15] was followed to evaluate the synergistic action of the LEO with an antibiotic. In our experimental protocol, the substance combinations were analysed by calculating the FIC index (FICI) using the following formulas:

(2) $$\mathrm{FIC}\;\mathrm{of}\;\mathrm{LEO}=\frac{\mathrm{MIC}\;\mathrm{value}\;\mathrm{of}\;\mathrm{combined}\;\mathrm{essential}\;\mathrm{oil}\;\mathrm{with}\;\mathrm{enrofloxacin}}{\mathrm{MIC}\;\mathrm{value}\;\mathrm{of}\;\mathrm{lavender}\;\mathrm{essential}\;\mathrm{oil}\;\mathrm{alone}}\;$$

(3) $$\mathrm{FIC}\;\mathrm{of}\;\mathrm{enrofloxacin}=\frac{\mathrm{MIC}\;\mathrm{value}\;\mathrm{of}\;\mathrm{combined}\;\mathrm{enrofloxacin}\;\mathrm{with}\;\mathrm{essential}\;\mathrm{oil}}{\mathrm{MIC}\;\mathrm{value}\;\mathrm{of}\;\mathrm{enrofloxacin}\;\mathrm{alone}}\;\;\;$$

(4) FICI=FIC value of essential oil +FIC value of enrofloxacin 

Generally, the FICI value was interpreted as: Synergistic when ≤0.5; additive when >0.5 and ≤1; noninteractive (>1 but ≤4) and antagonistic when >4 [14].

## 3. Statistical Analysis

### 3.1. Chicken Experiment

In this study, H_0_ hypothesis assumed that adding 0.4 mL/L LEO to the drinking water and its time of its application for chickens does not affect BW, ADG, FCR, FI, WI, EBI, survival rate and selected indices of blood serum. An alternative hypothesis—H_1_—assumes the occurrence of a significant effect on the considered variables. The obtained results of BW, ADG, FCR, FI, WI, EBI and selected indices of blood serum were subjected to statistical analysis, calculating the arithmetic means and standard error of the mean (SEM). The normality of the data was checked using the Shapiro–Wilk test. Residuals were normally distributed, and the difference between the groups were determined by analysis of variance (ANOVA) and post-hoc Tukey’s test. The performance parameters of broiler chickens of the three groups were tested with a two-way ANOVA to determine whether the observed factor (dietary treatment), and period (repeat measurement) had a significant impact on the measured parameter. In the event of a significant difference, a Tukey post hoc analysis was then performed to compare all groups with each other. The model includes an interaction test between these effects. The survival rate in the examined groups was compared with Fisher’s exact test. Biochemical, immunological and antioxidant indices in the blood serum of broiler chickens of the three groups were tested with a one-way ANOVA to determine whether the observed factor (dietary treatment) had a significant impact on the measured parameter. In the event of a significant difference, a Tukey post hoc multiple comparison test was performed to identify the differences among the means of the three groups. Differences were considered significant when *p* < 0.05. Statistical analysis was performed using the PQStat v. 1.6.2 package (PQStat Software, Plewiska, Poland).

### 3.2. In Vitro Experiment

The obtained results of antioxidant and antibacterial activity were subjected to calculating the arithmetic means and standard deviation. The tests were carried out in triplicate. The antioxidant and antibacterial activity were calculated using GraphPad Prism version 8.0 software.

## 4. Results

### 4.1. Chicken Experiment

Table 2 summarises the growth performance outcomes. The BW of one-day-old chicks was similar and ranged from 40 to 41 g. The addition of LEO to the potable water (*p* < 0.01) significantly improved the chickens’ 42 day of age, regardless of the application period. On the 42nd day of rearing, the highest BW was reported in the LEO_1–42_ group (2791 g), while the lowest BW was observed in the control group (2613 g)–(*p* < 0.01). The differences in the ADG revealed statistical significance in the second (day 22–42) and the whole (day 1–42) rearing period (*p* < 0.01). No differences were observed between the groups for the feed intake (FI) and water intake (WI) (*p* > 0.05). The daily FI was similar (105–106 g/d/bird). The daily WI (mL/d/bird) ranged from 219 in the LEO_22–42_ group to 229 in the control group. The application of LEO contributed positively to the feed conversion rate (FCR) in the second (day 22–42) and the entire rearing period (day 1–42). The highest, statistically significant FCR (*p* < 0.01) was observed in the control group (1.72 *g*/*g*), where no LEO was added. The FCR (*g*/*g*) ranged from 1.62 in the LEO_1–42_ group to 1.71 in the control group for the whole rearing period. The broiler chickens’ survival was similar in all groups and ranged from 97% in the control and LEO_1–42_ group to 98% in the LEO_22–42_ group (*p* > 0.05). The performance of broiler birds was also evaluated in terms of EBI, which includes ADG and survival percentage. Higher values of this indicator were observed in groups where LEO was added to the drinking water. The EBI amounted to 345 values in the control group and grew, reaching from 388 in LEO_22–42_ group to 396 values in the LEO_1–42_ group for the entire rearing period (day 1–42).

### 4.2. Serum Biochemical, Immunological and Antioxidant Indices

The results of the analysis of selected biochemical, immunological and antioxidant indices of the blood serum after day 42 of the experiment are summarised in Table 3. The addition of LEO to drinking water did not significantly affect the concentration of each biochemical index, which amounted to the following (mmol/L): Cholesterol (from 3.07 in the LEO_22–42_ group to 3.49 in the w LEO_1–42_ group), glucose (from 10.34 in the LEO_1–42_ group to 10.82 in the control group) and triglycerides (from 0.54 in the control group to 0.63 in the LEO_1–42_ group). The uric acid concentration (µmol/L) ranged from 287.6 in the control group to 325.2 in the LEO_1–42_ group. No differences in the serum’s total protein content were observed, whose concentration (g/L) ranged from 38.0 in the LEO_22–42_ group to 39.4 in the control group. The concentration of liver enzymes amounted to (U/L): Alkaline phosphatase (from 532.4 in the LEO_22–42_ group to 630.6 in the control group), alanine transaminase (from 19.4 in the control group to 20.60 in the LEO_22–42_ group) and aspartate aminotransferase (from 307.8 in the LEO_22–42_ group to 509.6 in the control group). No significant differences were observed for the immunological indices. The immunoglobulin concentration amounted to (mg/mL): IgG (from 10.21 in the control group to 12.40 in the LEO_1–42_ group), IgM (from 1.46 in the LEO_22–42_ group to 2.1 in the LEO_1–42_ group) and IgA (from 0.7 in the LEO_22–42_ group to 0.79 in the control group). Significant differences were revealed only for TAS (*p* < 0.01). The serum of chickens obtaining potable water with LEO, regardless of the administration period, was characterised by a higher TAS concentration than the control group; it ranged (mmol/L) from 1.55 (in the control group) to 2.26 (in the LEO_1–42_ group).

### 4.3. In Vitro Experiment

#### 4.3.1. Antioxidant Activity

**Figure 1:**
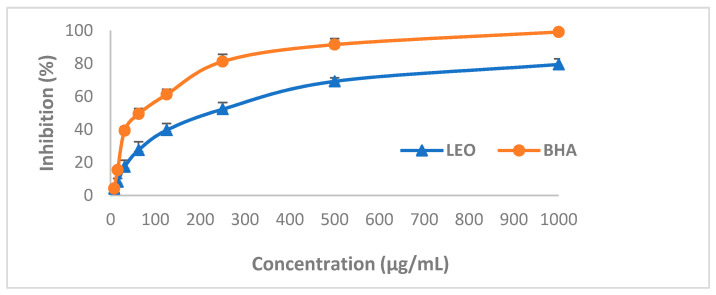
Antioxidant activity of lavender essential oil (LEO) and antioxidant standard 3-tert-butyl-4-hydroxyanisole (BHA).

Figure 1 showed the inhibition percentage of DPPH when the DPPH solution was tested against various LEO and BHA concentrations. LEO revealed antioxidant properties, which were increasing with its rising concentration. After 30 min of incubation, LEO neutralised the DPPH radical solution at 4.11–79.51% for concentrations of 7.81–1000 μg/mL, respectively. BHA activity, a substance with proven antioxidant action, amounted to 4.18–99.21%, respectively, in the same concentrations. The IC_50_ values of standard BHA and LEO were 61.1 μg/mL and and 216 μg/mL, respectively.

#### 4.3.2. Synergy Test: Microdilution Checkerboard Method

Table 4 shows the test results of LEO reactions combined with enrofloxacin with the checkerboard method. Individual interactions of LEO combined with enrofloxacin were diversified, from those suggesting synergism (*E. coli* 12 mm: LEO FICI—0.50; *E. coli* ≤ 6 mm: LEO FICI—0.22) through to an additive effect (*E. coli* ATCC 25922: LEO FICI—0.66; *E. coli* 30 mm: LEO FICI—1.0; *E. coli* 21 mm: LEO FICI—0.75; *E. coli* 17 mm: LEO FICI—0.56).

## 5. Discussion

The results of studies that aimed to evaluate the influence of EO addition to the diet on the growth performance parameters of poultry are not unequivocal [4,5,16,17,18,19]. A positive influence of LEO addition to the drinking water (0.5 mL/L) on the BW and FCR was obtained in the current experiment in the second (22–42 day) and the whole period (1–42 day) of LEO use (Table 2). Yarmohammadi Barbarestani et al. [5] obtained similar results in assessing the influence of LEO addition (main chemical ingredients: Linalool—38.12%; linalyl acetate—25.79%) to the feed at 600 mg/kg concentration. The study proved the efficacy of the influence on the chickens’ growth, mainly by an improvement of the intestinal microflora balance, intestine epithelium structure and antioxidant capability. Küçükyilmaz et al. [18] reported that EO feed with *Lavandula stoechas* (main chemical ingredients: Carvacrol—24.5%; 1.8-cineol—20.1%) had a negative impact on the BW, FI and FCR in the whole rearing period. Mokhtari et al. in their studies [16] discovered that an addition of LEO (main chemical ingredients: Linalool—44.31%; linalyl acetate—32.98%) to the feed at 100–800 mg/kg did not affect the fattening production parameters, while a reduction in the breast muscle weight was observed for the supplementation dose of 400 mg/kg feed. The diversification of the test results can be related to the applied supplementation, concentration, form, and administration method (water, feed) and the EOs’ ingredients’ biological activity. The EOs, owing to their activity against pathogenic microbes, contribute to the improvement of bacterial flora composition in the alimentary tract [4,5,16].

According to Mathlouthi et al. [19], the beneficial impact of active herb substances on the broiler chickens’ performance resulted from their ability to stimulate digestive processes and absorb nutrients and modulate the body’s immune system. The blood parameters are regarded as indices of the body’s health condition [17,20,21,22]. They help diagnose poultry diseases and provide essential knowledge in immunological tests and help evaluate the treatment’s efficacy [21]. EOs can be natural immunostimulators. Mathlouthi et al. [19] quoted that EOs and herb extracts can improve broiler chickens cellular and humoral immunity and reduce their susceptibility to infectious diseases. The EOs with hypercholesterolemic effect have a positive influence on health. Moreover, some of them may inhibit liver enzyme activity (HMG-CoA reductase) by regulating the endogenous cholesterol amount and reducing its concentration in the blood [22]. There are only a few literature papers on the influence of LEO on biochemical, immunological and antioxidant blood indices. Our tests on blood’s biochemical indices in broiler chickens revealed a lack of statistical differences between the groups (Table 3). The results correspond to the previous diet experiment [4] and the results obtained by Torki et al. [17], who did not demonstrate LEO supplementation’s influence on the blood biochemical parameters in laying hens. Mokhari et al. [16], who used 100, 200, 400, 600, and 800 LEO (mg/kg) as a feed additive, did not observe any differences in the total protein, and cholesterol, triglycerides, HDL, LDL or uric acid. They revealed a glucose concentration reduction in the groups of LEO-supplemented chickens compared to the control group. Yarmohammadi Barbarestania et al. [5], in their evaluation of LEO application in broiler chickens’ diets, did not observe any differences in glucose concentrations, total protein, triglycerides, HDL or VLDL. Among the tested biochemical indices, significant differences were revealed only for the cholesterol level—the supplementation with 600 mg/kg LEO greatly reduced the cholesterol level compared to the control group and the group supplemented with 300 mg/kg LEO. Abo Ghanima et al. [22] also discovered a decrease in the blood cholesterol level in the laying hens supplemented with the feed of rosemary and cinnamon oil (300 mg/kg feed). Moreover, the quoted authors demonstrated a reduction in the ALT and AST enzymes in the EOs supplemented groups. Some EOs and the chemical compounds in their ingredients stimulate the immune system. Once used in the diet, they improve the broiler chickens’ immunity, leading to a reduction in the broiler chickens’ susceptibility to infectious diseases [21]. Mohiti-Asli and Ghanaatparast-Rashti [21] revealed that EO with origanum at the dose of 300 ppm in the broiler chickens’ diet greatly improved the level of IgG immunoglobulins without affecting the IgM value. Gao et al. [23] reported that the EOs mixture they tested was a strong immunomodulating agent, which raised the (IgG, IgM) immunoglobulins level in laying hens. Mahrous et al. [24] discovered that the immunoglobulin level in the serum (IgA, IgG, and IgM) was significantly (*p* < 0.05) higher in broiler chickens whose feed contained 1.0% and 1.5% clove oil. Our experiment revealed that LEO application to the drinking water did not affect the level of the three antibodies described in the birds: IgA, IgM and IgG (Table 3). The results correspond to the results obtained by other researchers, including Aami-Azghadi et al. [25], who demonstrated that the inclusion of EOs from carraway and wormwood at 100, 200 and 300 ppm into the chickens’ diet did not increase the IgM and IgG immunoglobulins titre. In their studies, Alp et al. [26] did not reveal EO’s influence from oregano added to the diet on the IgG level in the chickens’ blood serum after day 42 of the experiment. The exact mechanism of EOs stimulation of the immunological response in broiler chickens and other animals is unknown, which is why further studies on the possibilities of EOs use in their diet are necessary. The TAS was also evaluated. An addition of 0.4 mL/L LEO significantly affected (*p* < 0.01) the TAS value. The TAS increase was observed in the blood serum of broiler chickens that received LEO in the initial rearing period (1–22 d) and in the whole rearing period (1–42 d), which confirmed its antioxidant properties. Ryzner et al. [27] also demonstrated a significant TAS increase in the chickens fed with *Satureja officinalis* essential oil at 0.05%. TAS means the body’s ability to defend itself against the action of free radicals, involving their inactivation to substances with a neutral load. The TAS values can be assumed to be a non-enzymatic antioxidant marker that reflects the body’s total volume, cell, tissue or organ’s antioxidant barrier. The parameter values can also measure the body’s pro-oxidant-antioxidant balance or the indicator of the antioxidant reserve exhausting in the course of any diseases that entail free oxygen radicals’ generation, e.g., colibacteriosis. Colibacteriosis is a cause of significant economic losses in poultry production. Infections caused by *E. coli* contribute to a high morbidity and mortality rate in poultry [1]. The infections are traditionally treated with fluoroquinols, e.g., enrofloxacin [28]. The application of enrofloxacin became a turning point in the treatment of poultry colibacteriosis, caused mainly by *O1*, *O2*, *O18* and *O78* serotypes [1]. Chemotherapeutics, initially highly effective in *E. coli* infection control, become ineffective after some time of their application due to the resistance developed by bacteria. The reported increase in the frequency of bacterial infections and resistance to the applied drugs forces the search for new solutions to improve the treatment’s efficacy. The use of EOs combined with chemotherapeutics is a promising study direction [29]. The fact that essential oils and chemotherapeutics are approved for use in animals benefit from combining antimicrobial substances, providing an advantage over newly synthesised chemicals whose launching requires long-term studies and significant financial outlays. The LEO reaction assessment, combined with enrofloxacin, was carried out in our study with the checkerboard method (Table 4). The calculated FICI values were used for demonstrating the effects of their combined reaction. By analysing the different enrofloxacin combinations with LEO, it was discovered that their effects on the test microbes were diversified. The important fact is that synergism of enrofloxacin and LEO action was achieved for *E. coli* resistant strains. In no cases was an antagonistic reaction observed. El Atki et al. [30] proved a synergistic action of cinnamon oil for *E. coli* ATCC 25,922 combined with chloramphenicol (FICI = 0.50). Other authors in their experiments demonstrated a diversified influence of other essential oils on *E. coli* strains [31,32]. It was also confirmed in own studies for LEO, whose combinations with enrofloxacin had not been previously investigated. The resistance of many bacteria strains to drugs, observed in recent decades, forced the use of combined treatments in contemporary medicine and veterinary. The antimicrobial action of EOs was empirically confirmed in many studies but their exact action mechanism has not been fully explained [3,29,33,34]. The synergistic reaction can result from the changes in the bacteria cell membrane permeability. Some EOs ingredients, e.g., carvacrol, which is abundant (>30%) in thyme oil, are characterised by the ability to permeabilise and depolarise the *E. coli* cytoplasm membrane, thus facilitating the antibiotic penetration into the bacteria cell [33]. The EOs were proven to influence the bacteria cell’s outer surface and cytoplasm. Hydrophobicity, typical of EOs, causes a rupture of bacteria structures, leading to improved permeability. The cell membrane permeability barrier is indispensable for many functions, including but not limited to maintaining the cells’ energy status, transport of dissolved substances and metabolic regulation. It suggests that EOs’ antimicrobial mechanisms can be related to the cell wall degradation, cytoplasm coagulation, improved permeability leading to the cell content leak and reduction in the protonomotoric force [3,34]. A few papers report a higher antibacterial activity of antibiotics used in combination with EOs in in vivo tests [35]. Studies on the synergistic action of EOs with antibiotics fit the strategy of combatting bacteria drug resistance.

## 6. Conclusions

The addition of LEO to the drinking water (0.4 mL/L) improved the production indices of broiler chickens, mainly due to the BW, ADG and EBI increase and FCR decrease. Additionally, no differences were observed between the periods of adding LEO (day 1–42 and day 22–42); thus, it may be assumed that adding LEO in the second period of rearing (day 22–42) is more profitable for economic reasons. LEO supplementation did not affect the blood serum biochemical and immunological indices, but it increased the TAS level, which is a testimony to its antioxidant properties. The inclusion of LEO as an additive to broiler chickens’ drinking water can become an excellent alternative to the forbidden antibiotic-based growth stimulators. Considering the demonstrated LEO’s in vitro synergy with enrofloxacin towards resistant *E. coli* strains, further studies are necessary to confirm the efficacy of the combination in in vivo tests.

## Figures and Tables

**Table 1:** Ingredient and nutrient composition of the basal diets (%).

Item	Starter (0–12 day)	Grower I (13–22 day)	Grower II (23–32 day)	Finisher (33–42 day)
Ingredient				
Wheat	37.00	38.00	33.00	40.00
Soybean meal	29.05	24.94	27.16	24.0
Maize	25.93	27.39	28.74	25.32
Soya oil	2.68	2.78	3.79	3.66
Canola meal	-	2.50	2.50	3.01
Potato protein	1.50	1.0	0.50	-
Limestone	1.27	0.85	0.78	0.67
Monocalcium phosphate	0.92	0.43	0.30	0.11
Vitamin and mineral premix ^1^	0.53	0.53	0.60	0.59
Poultry fat	-	0.50	1.50	1.50
L-Lys-HCl	0.39	0.40	0.41	0.43
DL-Met	0.26	0.17	0.18	0.17
Salt	0.24	0.27	0.28	0.28
NaHCO_3_	0.14	0.13	0.15	0.14
Threonine	0.07	0.06	0.07	0.07
Choline chloride	-	0.03	0.02	0.03
Phytase premix and coccidiostat ^2^	0.02	0.02	0.02	0.02
Calculated analysis				
ME ^3^ (kcal/kg)	2800	2865	2984	3020
Lys	1.27	1.17	1.15	1.07
Met	0.56	0.42	0.40	0.44
Ca	0.86	0.63	0.56	0.65
*p*	0.56	0.46	0.43	0.39
Na	0.15	0.15	0.15	0.15
Analysed nutrient composition ^4^				
Crude protein	21.05	19.47	19.40	18.52
Crude fibre	3.18	3.47	3.62	3.38
Crude fat	4.51	4.85	6.75	7.36
Crude ash	5.24	3.96	3.76	4.00

^1^ Vitamin–mineral premix contained the following per kilogram of diet: Vitamin A, 12,500 IU; vitamin D3, 5000 IU; vitamin E, 50 mg; vitamin B1, 3 mg; vitamin B2, 10 mg; vitamin B6, 3 mg; vitamin B12, 15 μg; nicotinic acid, 60 mg; pantothenic acid, 14.7 mg; folic acid, 1.5 mg; iron, 63 mg; copper, 15 mg; cobalt, 1.0 mg; zinc, 100 mg; iodine, 1.0 mg; selenium, 0.3 mg, antioxidant (BHA); ^2^ Phytase premix was prepared by dilution with calcium carbonate to contain 750 FTU (phytase units)/g (Optiphos, Huvepharma AD, Sofia, Bulgaria); Salinomicin sodium 70 mg/kg (Salinomax 120 G, Zoetis Belgium SA); ^3^ Metabolizable Energy; ^4^ Based on a DM content of 87.5%.

**Table 2:** The effect of LEO on the performance parameters of broiler chickens from 1 to 42 days of age ^1^.

Measurement Per Period ^3^ (day)	Dietary Treatment ^2^			SEM ^4^
	Control	LEO_1–42_	LEO_22–42_	
BW (g)				
1	40	40	41	0.14
21	1021	1028	1013	11.80
42	2612 ^a^	2791 ^b^	2751 ^b^	18.89
ADG (g/day)				
1–21	47	47	46	0.70
22–42	76 ^a^	84 ^b^	88 ^b^	0.15
1–42	61 ^a^	66 ^b^	65 ^b^	0.21
FI (g/day)				
1–21	58	61	59	0.25
22–42	171	176	172	1.72
1–42	105	106	105	0.46
FCR (g/g)				
1–21	1.25	1.30	1.28	0.02
22–42	2.26 ^a^	2.09 ^b^	2.08 ^b^	0.05
1–42	1.72 ^a^	1.62 ^b^	1.63 ^b^	0.04
WI (mL/day)				
1–21	143	149	145	1.07
22–42	351	350	353	2.88
1–42	229	224	219	1.02
EBI values				
1–21	364	354	353	1.51
22–42	325 ^a^	394 ^b^	411 ^b^	7.04
1–42	345 ^a^	396 ^b^	387 ^b^	8.12
*p*-value	Period	Treatment		Period × Treatment
BW	<0.01	<0.01		<0.01
ADG	<0.01	<0.01		<0.01
FI	<0.01	0.99		0.98
FCR	<0.01	<0.01		<0.01
WI	<0.01	0.97		0.98
EBI	<0.01	<0.01		<0.01

^a,b^—values in rows with different letters differ significantly (*p* < 0.01); ^1^ Results are means of 100 broiler chickens (five replicates of 20 broiler chicken) per treatment; ^2^ dietary treatment: control = no additive, LEO_1–42_ = addition of 0.4 mL/L LEO from day 1 to 42 day of age, LEO_22–42_ = addition of 0.4 mL/L LEO from day 22 to 42 day of age; ^3^ BW: body weight, ADG: average daily gain, FI: feed intake, FCR: feed conversion ratio, WI: water intake; EBI: European Broiler Index; ^4^ SEM: standard error of the mean.

**Table 3:** Biochemical, immunological and antioxidant indices in the blood serum of broiler chickens.

Blood Serum Parameters	Control	Group ^1^	LEO_22–42_	SEM ^2^	*p*-Value
		LEO_1–42_			
Alkaline phosphatase (U/L)	630.60	582.60	532.40	18.11	0.822
Alanine transaminase (U/L)	19.40	20.20	20.60	0.97	0.910
Aspartate aminotransferase (U/L)	509.60	402.00	307.80	19.61	0.134
Cholesterol (mmol/L)	3.30	3.49	3.07	0.18	0.442
Glucose (mmol/L)	10.82	10.34	10.65	0.51	0.401
Total protein (g/L)	39.40	38.80	38.00	1.21	0.873
Triglyceride (mmol/L)	0.54	0.63	0.57	0.08	0.361
Uric acid (μmol/L)	287.60	325.20	319.40	9.33	0.716
IgG ^3^(mg/mL)	10.21	12.40	11.91	0.62	0.164
IgM ^4^(mg/mL)	1.89	2.10	1.46	0.33	0.228
IgA ^5^(mg/mL)	0.79	0.78	0.70	0.08	0.791
Total antioxidant status (mmol/L)	1.55 ^a^	2.26 ^b^	2.22 ^b^	0.67	<0.001

^a,b^—values in rows with different letters differ significantly (*p* < 0.01);^1^ LEO_1–42_ = addition of 0.4 mL/L LEO from d 1 to 42 d of age, LEO_22–42_ = addition of 0.4 mL/L LEO from d 22 to 42 d of age; ^2^ SEM: standard error of the mean.; ^3^ Immunoglobulin G; ^4^ Immunoglobulin M; ^5^ Immunoglobulin A.

**Table 4:** Lavender essential oil (LEO) in combination with enrofloxacin—Fractional Inhibitory Concentration (FIC) and FIC Indices (FICI).

Item	MIC_0_	MIC_c_	FIC	FICI	Type of Interaction
*Escherichia coli* ATCC 25922 (*Susceptible*-35 mm) ^1^					
LEO (% *v*/*v*)	0.50	0.08	0.16	0.66	additive
Enrofloxacin (µg/mL)	0.01	0.005	0.50		
*Escherichia coli* (*Susceptible*-30 mm) ^1^					
LEO (% *v*/*v*)	1.0	0.75	0.75	1.0	additive
Enrofloxacin (µg/mL)	0.02	0.005	0.25		
*Escherichia coli* (*Intermediate*-21 mm) ^1^					
LEO (% *v*/*v*)	1.0	0.625	0.625	0.75	additive
Enrofloxacin (µg/mL)	0.320	0.04	0.125		
*Escherichia coli* (*Intermediate*-17 mm) ^1^					
LEO (% *v*/*v*)	1.0	0.50	0.50	0.56	additive
Enrofloxacin (µg/mL)	5.0	0.32	0.06		
*Escherichia coli* (*Resistant*-12 mm) ^1^					
LEO (% *v*/*v*)	1.0	0.50	0.50	0.50	synergistic
Enrofloxacin (µg/mL)	10.0	0.04	0.004		
*Escherichia coli* (*Resistant* ≤ 6 mm) ^1^					
LEO (% *v*/*v*)	1.0	0.16	0.16	0.22	synergistic
Enrofloxacin (µg/mL)	40.0	2.50	0.062		

^1^—sensitivity to enrofloxacin with the growth inhibition zone using Enrofloxacin antibiotic disc Enrofloxacin ENO-5 (BD BBL™ Sensi Disc; Becton Dickinson, MD, USA) ± 1 mm; MIC—Minimal Inhibitory Concentration; FIC—Fractional Inhibitory Concentration; FICI—Fractional Inhibitory Concentration Index; MIC_0_ = MIC of an individual sample alone; MICc = MIC of an individual sample of the most effective combination; FIC of essential oil = MIC of essetial oil in combination with enrofloxacin/MIC of essential oil alone; FIC of enrofloxacin = MIC of enrofloxacin in combination with essential oil/MIC of enrofloxacin alone; FIC index = FIC of essential oil + FIC of enrofloxacin; FICI ≤ 0.5, synergistic; FICI > 0.5–1.0, additive; FICI > 1.0–4.0, no interaction; FICI > 4.0, antagonistic.

## Data Availability

Not applicable.
